# Supplementary material for: Projected entangled pair states for lattice gauge theories with dynamical fermions
Source: Commun Phys. 2026 Jan 8;9(1):50. doi: 10.1038/s42005-025-02482-7 (PMC12890585; doi:10.1038/s42005-025-02482-7)
Supplement: Supplementary file 1 — Supplementary Material [file 42005_2025_2482_MOESM1_ESM.pdf]

Projected Entangled Pair States for Lattice Gauge Theories with Dynamical Fermions Supplementary Information

Ariel Kelman ,[1,*] Umberto Borla ,[1,2,3] Patrick Emonts ,[4,5,6,7,†] and Erez Zohar [1,8]

[1] *Racah Institute of Physics, The Hebrew University of Jerusalem, Givat Ram, Jerusalem 91904, Israel*
[2] *Max Planck Institute of Quantum Optics, 85748 Garching, Germany*
[3] *Munich Center for Quantum Science and Technology (MCQST), 80799 Munich, Germany*
[4] *Institute for Complex Quantum Systems, Ulm University, 89069 Ulm, Germany*
[5] *Center for Integrated Quantum Science and Technology (IQST), Ulm-Stuttgart, Germany*
[6] *Instituut-Lorentz, Universiteit Leiden, Niels Bohrweg 2, 2333 CA Leiden, Netherlands*
[7] *$\langle aQa^L \rangle$ Applied Quantum Algorithms, Universiteit Leiden*
[8] *School of Physics and Astronomy, Tel Aviv University, Tel Aviv 6997801, Israel*

(Dated: December 22, 2025)

Supplementary Information A: Ansatz Details

In the main text, we focused on the most salient points of the ansatz — this section gives a more complete description and adds to the presentation in the main text. We start with a discussion of the number of virtual modes used in the construction. It was mentioned in the Ansatz section of the main text that the particular ansatz utilized here uses four copies of virtual modes per site per link. It was shown in [1] that for a pure gauge $\mathbb{Z}_2$ theory, in order for the ansatz to be expressive enough to capture the ground state limits of the (pure gauge) Hamiltonian, at least two copies are needed. For the ansatz which includes physical matter, it would *prima facie* be unnecessary to add in additional virtual modes. However, the inclusion of matter requires the consideration of a global $U(1)$ symmetry. To ensure that the constructed ansatz state is invariant under this symmetry, we require virtual modes that are charged under the symmetry. Thus, each virtual mode must fall into one of three categories [2]:

1. Those which are uncharged under the global $U(1)$ symmetry.
2. Those which are charged under the global $U(1)$ symmetry in the same way as the matter.
3. Those which are charged under the global $U(1)$ symmetry in a complementary way (i.e. conjugate) to the matter, and so pick up the opposite phase to the matter.

Note that after the particle hole transformation, the $U(1)$ symmetry acts differently on physical modes on the even/odd sublattices.. A given copy of the virtual modes will similarly transform with opposite phases under the $U(1)$ transformation on the even/odd sublattices. As a result of the $U(1)$ symmetry, modes — physical or virtual — can only couple to modes which pick up the opposite phase. In order to allow our ansatz to be at least as expressible as the one considered in [1] — which allowed all-to-all coupling of the modes, since they were all of the first type — we therefore leave two copies of virtual modes to play the same role, and leave them uncharged by the $U(1)$ symmetry. This ensures that the ansatz can capture the pure-gauge physics of the system. To accommodate virtual modes which couple to physical matter, we must therefore add a third copy. This is insufficient, however, since one copy would not allow for virtual-virtual coupling of modes on the same site as is done in the operator $A(\mathbf{x})$. We therefore include a copy of each of the latter two types, bringing the total to four.

Once the number of virtual modes is specified, it remains to be shown how they transform under the symmetries of the state, and the constraints this imposes on the construction of the ansatz. The modes transform under the $U(1)$ symmetry as just described — copies 1 and 2 are uncharged, copy 3 transforms with the opposite phase of the physical modes, while copy 4 transforms in the same way as the physical modes. Thus copies 1 and 2 can couple freely to each other, but not to copies 3, 4, or the physical modes. Copy 3 can only couple to copy 4 modes belonging to the same site as well as the physical mode there, or — since neighboring sites are never on the same even/odd sublattice — to copy 3 modes of neighboring sites, as happens in the projector operators.

Therefore, the structure of $\mathcal{T}$, which couples the various modes in the operator A, is

$$\begin{array}{c} \\ \mathcal{T} = \end{array} \begin{array}{c} \begin{array}{ccccc} \psi & \text{copy 1} & \text{copy 2} & \text{copy 3} & \text{copy 4} \end{array} \\ \left(\begin{array}{c|cc:cc} 0 & \mathbf{0} & \mathbf{0} & M & \mathbf{0} \\ \hline \mathbf{0} & \multicolumn{2}{c:}{\multirow{2}{*}{V^{PG}}} & \mathbf{0} & \mathbf{0} \\ \mathbf{0} & & & \mathbf{0} & \mathbf{0} \\ \hdashline -M^{\top} & \mathbf{0} & \mathbf{0} & \mathbf{0} & \tilde{V} \\ \mathbf{0} & \mathbf{0} & \mathbf{0} & -\tilde{V}^{\top} & \mathbf{0} \end{array}\right) \end{array}, \quad \text{(A1)}$$

where the blocks have further structure in order to guarantee the other symmetries, as discussed below. Note that the gauging operators $\mathcal{U}$ and projectors w only couple allowed modes, and so this completes accounting for the constraints arising from the $U(1)$ symmetry. We have also taken into account the canonical fermionic anticommutation relations.

* ariel.kelman@mail.huji.ac.il; Corresponding author.
† patrick.emonts@uni-ulm.de; Corresponding author.

In order to ensure translation invariance, it is sufficient to require that $\mathcal{T}(\mathbf{x}) = \mathcal{T}$. This was already indicated by the suppression of site dependence in equation (A1). Ensuring translation invariance does not impose any extra structure on the gauging or projector operators.

Finally, we consider invariance under rotations. We introduce the permutation matrix

$$\mathcal{R}_0 = \begin{pmatrix} 0 & 1 & 0 & 0 \\ 0 & 0 & 1 & 0 \\ 0 & 0 & 0 & 1 \\ 1 & 0 & 0 & 0 \end{pmatrix}, \tag{A2}$$

which rotates a single copy of the virtual modes around a site (ordered right, up, left, down). If we group the creation operators of all of the modes of a given site into a vector, the rotation is implemented by

$$\mathcal{R} = \left(\begin{array}{c|ccc} \eta & & & \\ \hline & \eta_\mu R_0 & & \\ & & \ddots & \\ & & & \eta_{\mu'} R_0 \end{array}\right), \tag{A3}$$

where there are four copies of $\mathcal{R}_0$ along the diagonal. Modes are ordered: physical, copy 1, 2, 3, 4, with the modes in each copy ordered: right, up, left, down. The phases η_μ are all taken to be $e^{i\pi/4}$, i.e. the same as η. This is a choice, but one that is consistent with the construction of the ansatz.

Inspection reveals that the gauging and projecting operators are invariant under such rotations (the operators switch which link they act on, but retain their form). It therefore remains to show that A remains invariant, for which we require that

$$\mathcal{R}^\top \mathcal{T} \mathcal{R} = \mathcal{T}. \tag{A4}$$

Imposing this condition gives structure to the submatrices in $\mathcal{T}$ shown in equation (A1). The elements which couple the first two copies — "PG" denotes "pure gauge" to indicate that no physical matter is included — have the form

$$V^{\mathrm{PG}} = \begin{array}{c} \begin{matrix} r_1 & u_1 & l_1 & d_1 & r_2 & u_2 & l_2 & d_2 \end{matrix} \\ \begin{pmatrix} 0 & -z_1 & -iy_1 & -iz_1 & ia & ib & ic & id \\ z_1 & 0 & -iz_1 & y_1 & -d & -a & -b & -c \\ iy_1 & iz_1 & 0 & z_1 & -ic & -id & -ia & -ib \\ iz_1 & -y_1 & -z_1 & 0 & b & c & d & a \\ -ia & d & ic & -b & 0 & -z_2 & -iy_2 & -iz_2 \\ -ib & a & id & -c & z_2 & 0 & -iz_2 & y_2 \\ -ic & b & ia & -d & iy_2 & iz_2 & 0 & z_2 \\ -id & c & ib & -a & iz_2 & -y_2 & -z_2 & 0 \end{pmatrix} \end{array}, \tag{A5}$$

while the copies which do couple to physical matter are given by

$$\tilde{V} = \begin{pmatrix} if & ig & ih & ik \\ -k & -f & -g & -h \\ -ih & -ik & -if & -ig \\ g & h & k & f \end{pmatrix}, \tag{A6}$$

and finally,

$$M = \begin{pmatrix} it & -t & -it & t \end{pmatrix}. \tag{A7}$$

All of the parameters are complex-valued.

The projectors defined in equations the main text also satisfy all of the symmetries just mentioned. Note that the projectors are here defined as the Hermitian conjugate of those given in [1].

Since modes of copies 1-2 never couple to modes of copies 3-4, the state can be factored into $\psi_I(\mathcal{G})$ and $|\psi_{II}(\mathcal{G})\rangle$ as described above. All of the calculations, including of covariance matrices, can therefore be done independently, which lowers the maximum dimension of the matrices that must be dealt with [2].

Supplementary Information B: Observables

In this section we show how to calculate observables for a fixed gauge field configuration from covariance matrices, which can be found as described in [2]. It was shown in [1] how to evaluate the operators on the gauge fields, including the magnetic and electric energies as well as their gradients. The calculation of the electric energy is described using the projectors of the pure gauge layer. To adapt the calculation to account for the different projectors used in the construction of $|\psi_{II}(\mathcal{G})\rangle$, one can follow the same procedure, with the indices appropriate to the modes used in the definition of the projectors in the layers with matter. Here we demonstrate how to evaluate terms involving matter. All results in this section are after the particle hole transformation of the Particle-Hole section of the main text.

We start by defining Majorana modes, as the entries in the resulting covariance matrix are purely real. They are defined in terms of the Dirac modes as

$$\begin{aligned} c^\dagger &= \frac{1}{2}(\gamma^{(1)} + i\gamma^{(2)}) & \gamma^{(1)} &= c + c^\dagger \\ c &= \frac{1}{2}(\gamma^{(1)} - i\gamma^{(2)}) & \gamma^{(2)} &= i(c - c^\dagger) \end{aligned} \tag{B1}$$

where $c^\dagger, c$ are Dirac creation and annihilation operators, and $\gamma^{(1)}, \gamma^{(2)}$ are the corresponding Majorana modes.

The covariance matrix of a state $|\psi(\mathcal{G})\rangle$ is then defined as

$$\begin{aligned} \Gamma_{\mathbf{x}^i,\mathbf{y}^j}(\mathcal{G}) &= \frac{i}{2}\langle[\gamma^{(i)}(\mathbf{x}), \gamma^{(j)}(\mathbf{y})]\rangle \\ &= \frac{i}{2}\frac{\langle\psi(\mathcal{G})|\,[\gamma^{(i)}(\mathbf{x}), \gamma^{(j)}(\mathbf{y})]\,|\psi(\mathcal{G})\rangle}{\langle\psi(\mathcal{G})|\psi(\mathcal{G})\rangle}, \end{aligned} \tag{B2}$$

and, if the state is Gaussian (as our ansatz is, for a fixed $\mathcal{G}$), this contains all the information required to reconstruct the state.

It remains to show how to calculate the mass and interaction terms of the Hamiltonian. We start by rewriting

the expectation value of the mass term of the Hamiltonian as

$$
\begin{aligned}
\langle H_{\rm M}\rangle &= \frac{\langle\psi|\, H_{\rm M}\, |\psi\rangle}{\langle\psi|\psi\rangle} \\
&= \sum_{\mathcal{G},\mathcal{G}'} \frac{\langle\mathcal{G}'|\, \langle\psi_{II}(\mathcal{G}')|\, \psi_I^*(\mathcal{G}') H_{\rm M} \psi_I(\mathcal{G})\, |\psi_{II}(\mathcal{G})\rangle\, |\mathcal{G}\rangle}{\langle\psi|\psi\rangle} \\
&= \sum_{\mathcal{G}} \frac{\langle\psi_{II}(\mathcal{G})|\, H_{\rm M}\, |\psi_{II}(\mathcal{G})\rangle}{\langle\psi_{II}(\mathcal{G})|\psi_{II}(\mathcal{G})\rangle} p(\mathcal{G})
\end{aligned} \tag{B3}
$$

using the probability defined in the main text and where $|\psi(Q)\rangle = \psi_I(Q)\,|\psi_{II}(Q)\rangle$. This is only possible because $H_{\rm M}$ only acts on the Hilbert space of $|\psi_{II}\rangle$. We define

$$
\mathcal{F}_{\rm M}(\mathcal{G}) = \frac{\langle\psi_{II}(\mathcal{G})|\, H_{\rm M}\, |\psi_{II}(\mathcal{G})\rangle}{\langle\psi_{II}(\mathcal{G})|\psi_{II}(\mathcal{G})\rangle} \tag{B4}
$$

which can be written in terms of the Majorana covariance matrix as

$$
\mathcal{F}_{\rm M}(\mathcal{G}) = \frac{1}{2}\sum_{\mathbf{x}} \Big(1 + \Gamma_{\mathbf{x}^2\mathbf{x}^1}(\mathcal{G})\Big) \tag{B5}
$$

where $\mathbf{x}^i$ indicates the index corresponding to the $i^{\rm th}$ Majorana mode on site $\mathbf{x}$. To derive this result, rewrite $H_{\rm M}$ as

$$
H_{\rm M} = \sum_{\mathbf{x}} \frac{1}{2}\Big(1 + [\psi^\dagger(\mathbf{x}), \psi(\mathbf{x})]\Big) \tag{B6}
$$

using the anticommutation relation $\{\psi^\dagger(\mathbf{x}), \psi(\mathbf{x})\} = 1$, and then write $\psi^\dagger(\mathbf{x}), \psi(\mathbf{x})$ in terms of Majorana modes. The result gives the elements of the covariance matrix as in equation (B5). The full mass energy is thus given by

$$
\langle H_{\rm M}\rangle = \sum_{Q} \left[\frac{1}{2}\sum_{\mathbf{x}} \Big(1 + \Gamma_{\mathbf{x}^2\mathbf{x}^1}(\mathcal{G})\Big)\right] p(\mathcal{G}). \tag{B7}
$$

A similar procedure allows for the evaluation of the interaction energy. The interaction energy can be calculated similarly (as was done in the case of the mass energy in equations (B3) and (B4)), since

$$
\begin{aligned}
\langle H_{\rm I}\rangle &= \frac{\langle\psi|\, H_{\rm I}\, |\psi\rangle}{\langle\psi|\psi\rangle} \\
&= \sum_{\mathcal{G},\mathcal{G}'} \frac{\langle\mathcal{G}'|\, \langle\psi_{II}(\mathcal{G}')|\, \psi_I^*(\mathcal{G}') H_{\rm I} \psi_I(\mathcal{G})\, |\psi_{II}(\mathcal{G})\rangle\, |\mathcal{G}\rangle}{\langle\psi|\psi\rangle} \\
&= \sum_{\mathcal{G}} \frac{|\psi_I(\mathcal{G})|^2\, \langle\mathcal{G}|\, \langle\psi_{II}(\mathcal{G})|\, H_{\rm I}\, |\psi_{II}(\mathcal{G})\rangle\, |\mathcal{G}\rangle}{\langle\psi|\psi\rangle} \\
&= \sum_{\mathcal{G}} \frac{\langle\mathcal{G}|\, \langle\psi_{II}(\mathcal{G})|\, H_{\rm I}\, |\psi_{II}(\mathcal{G})\rangle\, |\mathcal{G}\rangle}{\langle\psi_{II}(\mathcal{G})|\psi_{II}(\mathcal{G})\rangle} p(\mathcal{G}),
\end{aligned} \tag{B8}
$$

so we define

$$
\mathcal{F}_{\rm I}(\mathcal{G}) = \frac{\langle\mathcal{G}|\, \langle\psi_{II}(\mathcal{G})|\, H_{\rm I}\, |\psi_{II}(\mathcal{G})\rangle\, |\mathcal{G}\rangle}{\langle\psi_{II}(\mathcal{G})|\psi_{II}(\mathcal{G})\rangle}. \tag{B9}
$$

To evaluate this expression, note that for a given gauge field configuration $\mathcal{G}$, in the magnetic basis

$$
\langle U(\mathbf{x}, k)\rangle = e^{i\delta q(\mathbf{x},1)} \tag{B10}
$$

where $\delta = \pi$, $q(\mathbf{x}, 1)$ is the value that $\mathcal{G}$ assigns to the link $\ell = (\mathbf{x}, k)$, and

$$
\begin{aligned}
\langle\psi^\dagger(\mathbf{x})\psi^\dagger(\mathbf{y})\rangle &= \frac{1}{4i}\Big(\Gamma_{\mathbf{x}^1\mathbf{y}^1} + i\Gamma_{\mathbf{x}^1\mathbf{y}^2} + i\Gamma_{\mathbf{x}^2\mathbf{y}^1} - \Gamma_{\mathbf{x}^2\mathbf{y}^2}\Big)_{\mathcal{G}} \\
\langle\psi(\mathbf{x})\psi(\mathbf{y})\rangle &= \frac{1}{4i}\Big(\Gamma_{\mathbf{x}^1\mathbf{y}^1} - i\Gamma_{\mathbf{x}^1\mathbf{y}^2} - i\Gamma_{\mathbf{x}^2\mathbf{y}^1} - \Gamma_{\mathbf{x}^2\mathbf{y}^2}\Big)_{\mathcal{G}}
\end{aligned} \tag{B11}
$$

using the same procedure involving commutators and Majorana modes used to evaluate the mass term. When this expression is used below, the expectation will be taken relative to the state $|\psi_{II}(\mathcal{G})\rangle$, and so all covariance matrices depend on $\mathcal{G}$, though this was suppressed to declutter the notation ($\psi_I(\mathcal{G})$ does not contribute, as can be seen from its absence in equation (B9), and as expected due the to absence of fermionic matter in $\psi_I(\mathcal{G})$).

Thus, the expectation of the interaction energy is given by

$$
\begin{aligned}
\langle H_{\rm I}\rangle = \sum_{\mathcal{G}} \Bigg[\sum_{\mathbf{x}} \frac{1}{2}\Big(& e^{i\pi q(\mathbf{x},1)} \big[\Gamma_{\mathbf{x}^1,(\mathbf{x}+\hat{\mathbf{e}}_1)^1} - \Gamma_{\mathbf{x}^2,(\mathbf{x}+\hat{\mathbf{e}}_1)^2}\big] \\
& - e^{i\pi q(\mathbf{x},2)} \big[\Gamma_{\mathbf{x}^1,(\mathbf{x}+\hat{\mathbf{e}}_2)^2} + \Gamma_{\mathbf{x}^2,(\mathbf{x}+\hat{\mathbf{e}}_2)^1}\big]\Big)\Bigg]_{\mathcal{G}} p(\mathcal{G})
\end{aligned} \tag{B12}
$$

where the large bracketed term is $\mathcal{F}_{\rm I}(\mathcal{G})$.

Note that as explained in [2], the action of any observable can be separated into its action on the various layers of the ansatz — in our case $\psi_I(\mathcal{G})$ and $|\psi_{II}(\mathcal{G})\rangle$.

To calculate the derivatives of the mass and interaction energies with respect to the parameters of the ansatz, it is sufficient to find the derivative of the covariance matrix of the state, which can be done using the expression for the covariance matrix found in [2].

Supplementary Information C: Free Fermion Limit

We now consider the free fermions limit of our model, which obtains when only the interaction coupling of the Hamiltonian is nonzero. This provides a simple benchmark for the ansatz for large systems (6×6 and larger). It further provides an indication of when finite size effects are significant in our model.

In the absence of electric coupling ($g_E = 0$), the gauge fields enter the Hamiltonian only through the operator $U = \sigma^x$, and can therefore be regarded as a static background. In this section we also set $g_M = g_B = 0$, so that the fermions are massless and no flux configuration is *a priori* preferred. The phases in the interaction (hopping) term give rise to a π-flux per plaquette, which results in a Dirac-like band structure and guarantees the correct continuum limit from the high-energy physics perspective. While a background $\mathbb{Z}_2$ gauge field can in principle

modify the flux by adding a π phase to any of the hoppings, this is guaranteed not to happen by Lieb's theorem [3]. This states that at half filling, the energetically preferred hopping configuration for free fermions on the square lattice is the one realizing a π-flux per plaquette.

The Hamiltonian including just the interaction energy is diagonal in momentum space, with a dispersion relation

$$E(k_x, k_y) = \pm 2 g_I \sqrt{\sin^2 k_x + \sin^2 k_y}, \qquad \text{(C1)}$$

which exhibits Dirac cones at $\vec{\mathbf{k}} = (0, 0)$ as expected. In position space the system has a 2×2 unit cell, leading to a reduced Brillouin zone. Both k_x and k_y are restricted to take values between 0 and π, and the points (k_x, k_y) and $(k_x+\pi, k_y+\pi)$ are identified with each other. On a torus of size $L_x = L_y = L$ the momentum in either direction is quantized in units of $2\pi/L$. Depending on whether periodic or anti-periodic boundary conditions are imposed on the fermionic wave-function, it can take the values

$$k_n = \left\{ \frac{2\pi n}{L} \right\} \qquad \text{(PBC)} \qquad \text{(C2)}$$

or

$$k_n = \left\{ \frac{2\pi(n+1/2)}{L} \right\} \qquad \text{(ABC)} \qquad \text{(C3)}$$

respectively, with $n \in \{0, 1, \ldots L-1\}$. We need to account for both possibilities and see which one provides the lowest energy, corresponding to the true ground state of the system. This is obtained by filling the energy bands (C1) with the available momentum states starting from the bottom, located at $k_x = k_y = \pi/2$, up to $E = 0$ where the Dirac cones touch. The results are summarized in supplementary table I, from which it results that the lowest energy always corresponds to anti-periodic boundary conditions. The ansatz described in the main text matches these predictions closely even in the computationally challenging case of a 6×6 system.

	Energy (PBC)	Energy (ABC)	GGPEPS
$L = 2$	0	$-4\sqrt{2}$ ≈ -5.6569	-5.6569
$L = 4$	$-4(2+\sqrt{2})$ ≈ -13.6569	-16	-15.958
$L = 6$	$-8(\sqrt{3}+\sqrt{6})$ ≈ -33.4523	$-4(3\sqrt{2}+2\sqrt{5})$ ≈ -34.8591	-34.353

Supplementary Table I. Ground state energies of the Hamiltonian including just the interaction term on tori of size $L_x = L_y = L$ for periodic (left) and anti-periodic (right) boundary conditions for the fermions. The true ground state always corresponds to anti-periodic boundary conditions.

For larger systems, the ground state energy of the free fermions case is shown in supplementary figure 1. In addition to providing a check on the GGPEPS numerical results for larger systems, this may also provide an indication for when finite-size effects are significant. While it is possible that finite-size effects are stronger for observables other than the energy, two considerations alleviate this concern: (i) as shown in figure 4 of the main text, our state accurately captures each term in the Hamiltonian, showing that our optimization captures the underlying physics and not solely the total energy; (ii) as shown in figure 7 of the main text, even at the lattice sizes considered here, we are able to observe the behaviour of observables in different regimes.

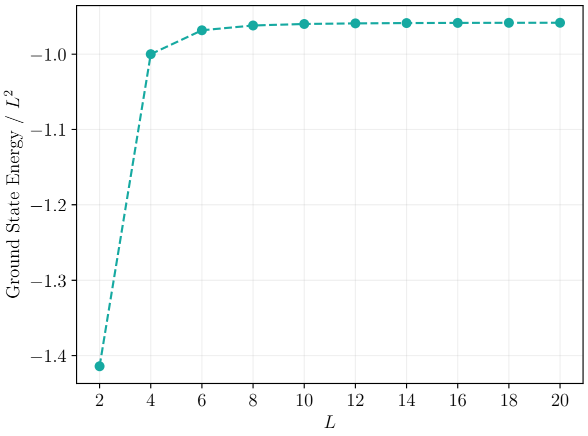

Supplementary Figure 1. The ground state energies, normalized by lattice size, for the free fermion Hamiltonian. The dashed lines are simply guides for the eye. Even for moderate lattice sizes, the normalized ground state energy is relatively close to its value in the thermodynamic limit.

Supplementary Information D: Computation Details

In this section, we provide some technical details on the configuration of our minimization scheme as well as Monte Carlo. Not all runs were run with identical settings, particularly as some points did not converge well on a first attempt, and were therefore rerun for further optimization.

The minimization algorithm used was BFGS, using the standard implementation of `scipy`, available through `scipy.optimize.minimize` with the default settings [4].

The number of Monte Carlo thermalization (warmup) steps was 50000 – 100000 (starting with the latter figure, but moving to the former as experiments confirmed that this was sufficient). The number of measurement steps was generally 120000. At each Monte Carlo step, some number of links were chosen to have their gauge fields randomly modified. This update size was 8 for the 4×4 system, and 20 for the 6×6 system.

SUPPLEMENTARY REFERENCES

[1] P. Emonts, A. Kelman, U. Borla, S. Moroz, S. Gazit, and E. Zohar, Finding the ground state of a lattice gauge theory with fermionic tensor networks: A $2+1$d $\mathbb{Z}_2$ demonstration, Physical Review D **107**, 014505 (2023).

[2] A. Kelman, U. Borla, I. Gomelski, J. Elyovich, G. Roose, P. Emonts, and E. Zohar, Gauged gaussian projected entangled pair states: A high dimensional tensor network formulation for lattice gauge theories, Phys. Rev. D **110**, 054511 (2024).

[3] E. H. Lieb, Flux phase of the half-filled band, Physical review letters **73**, 2158 (1994).

[4] P. Virtanen, R. Gommers, T. E. Oliphant, M. Haberland, T. Reddy, D. Cournapeau, E. Burovski, P. Peterson, W. Weckesser, J. Bright, S. J. van der Walt, M. Brett, J. Wilson, K. J. Millman, N. Mayorov, A. R. J. Nelson, E. Jones, R. Kern, E. Larson, C. J. Carey, İ. Polat, Y. Feng, E. W. Moore, J. VanderPlas, D. Laxalde, J. Perktold, R. Cimrman, I. Henriksen, E. A. Quintero, C. R. Harris, A. M. Archibald, A. H. Ribeiro, F. Pedregosa, P. van Mulbregt, and SciPy 1.0 Contributors, SciPy 1.0: Fundamental Algorithms for Scientific Computing in Python, Nature Methods **17**, 261 (2020).
